# *MYH7* p.(Arg1712Gln) is pathogenic founder variant causing hypertrophic cardiomyopathy with overall relatively delayed onset

**DOI:** 10.1007/s12471-023-01798-9

**Published:** 2023-07-24

**Authors:** Luisa Marsili, Freyja H. M. van Lint, Francesco Russo, Karin Y. van Spaendonck-Zwarts, Flavie Ader, Marie-Line Bichon, Laurence Faivre, Arjan C. Houweling, Bertrand Isidor, Ronald H. Lekanne Deprez, Moniek G. P. J. Cox, Arthur A. M. Wilde, Benoit Mazel, Sandra Mercier, Dennis Dooijes, Gilles Millat, Karine Nguyen, Jan G. Post, Pascale Richard, Irma van de Beek, Alexa M. C. Vermeer, Ludolf Boven, Jan D. H. Jongbloed, J. Peter van Tintelen, The European Reference Network for Rare and Low Prevalence Complex Diseases of the Heart, Luisa Marsili, Freyja H. M. van Lint, J. Peter van Tintelen, Arjan C. Houweling, Ronald H. Lekanne Deprez, Arthur A. M. Wilde, Dennis Dooijes, Jan G. Post, Irma van de Beek, Alexa M. C. Vermeer, Karin Y. van Spaendonck-Zwarts, Flavie Ader, Pascale Richard, Bertrand Isidor, Marie-Line Bichon, Sandra Mercier

**Affiliations:** 1 Clinique de génétique Guy Fontaine, CHU Lille, 59000 Lille, France; 2 Department of Genetics, University Medical Centre Utrecht, Utrecht University, Utrecht, The Netherlands; 3 Department of Human Genetics, Radboud University Medical Centre, Nijmegen, The Netherlands; 4 Service de Biochimie Métabolique, Hôpital Universitaire Pitié Salpêtrière, APHP—Sorbonne Université—DMU BioGem-Unité Fonctionnelle de Cardiogénétique et Myogénétique Moléculaire et cellulaire, 75651 Paris, France; 5 INSERM UMRS1166 Equipe 1, ICAN institute (institut de cardiométabolisme et nutrition), 91 Bd de l’hôpital, 75013 Paris, France; 6 UFR de Pharmacie, Université Paris Cité, 4 av de l’observatoire, 75006 Paris, France; 7 Department of Human Genetics, Amsterdam University Medical Centres, location Academic Medical Centre/University of Amsterdam, Amsterdam, The Netherlands; 8 Department of Cardiology, Amsterdam University Medical Centres, location Academic Medical Centre/University of Amsterdam, Amsterdam, The Netherlands; 9 Department of Cardiology, University Medical Centre Groningen, Groningen, The Netherlands; 10 Department of Genetics, University Medical Centre Groningen, Groningen, The Netherlands; 11 Centre de Génétique, FHU TRANSLAD—CHU Dijon Bourgogne, Dijon, France; 12 Service de Génétique Médicale, CHU de Nantes, Nantes, France; 13 Unité Fonctionnelle de Cardiogénétique Moléculaire, LBMMS, Centre de Biologie et Pathologie Est, Hospices Civils de Lyon, 69677 Bron, France; 14 Université de Lyon 1, Lyon, France

**Keywords:** Founder mutation, Myosin heavy chain 7, MYH7, Cardiomyopathy, Hypertrophic cardiomyopathy

## Abstract

**Introduction:**

The *MYH7* c.5135G > A p.(Arg1712Gln) variant has been identified in several patients worldwide and is classified as pathogenic in the ClinVar database. We aimed to delineate its associated phenotype and evaluate a potential founder effect.

**Methods:**

We retrospectively collected clinical and genetic data of 22 probands and 74 family members from an international cohort.

**Results:**

In total, 53 individuals carried the *MYH7* p.(Arg1712Gln) variant, of whom 38 (72%) were diagnosed with hypertrophic cardiomyopathy (HCM). Mean age at HCM diagnosis was 48.8 years (standard deviation: 18.1; range: 8–74). The clinical presentation ranged from asymptomatic HCM to arrhythmias (atrial fibrillation and malignant ventricular arrhythmias). Aborted sudden cardiac death (SCD) leading to the diagnosis of HCM occurred in one proband at the age of 68 years, and a family history of SCD was reported by 39% (5/13) probands. Neither heart failure deaths nor heart transplants were reported. Women had a generally later-onset disease, with 14% of female carriers diagnosed with HCM at age 50 years compared with 54% of male carriers. In both sexes, the disease was fully penetrant by age 75 years. Haplotypes were reconstructed for 35 patients and showed a founder effect in a subset of patients.

**Conclusion:**

*MYH7 p*.(Arg1712Gln) is a pathogenic founder variant with a consistent HCM phenotype that may present with delayed penetrance. This suggested that clinical follow-up should be pursued after the seventh decade in healthy carriers and that longer intervals between screening may be justified in healthy women < 30 years.

**Supplementary Information:**

The online version of this article (10.1007/s12471-023-01798-9) contains supplementary material, which is available to authorized users.

## What’s new?

- A large series of patients with the *MYH7* p.(Arg1712Gln) variant showed: (1) a consistent hypertrophic cardiomyopathy (HCM) phenotype, (2) women developing HCM at a later age, and (3) development of HCM after the sixth decade.
- Describing a series of individuals with a single pathogenic variant is important to establish variant-specific screening protocols to prevent unnecessary cardiac evaluations.
- For healthy *MYH7* p.(Arg1712Gln) carriers, longer screening intervals could be appropriate for women younger than 30 years and follow-up should be continued after the age of 60 years.
- Our data also showed that *MYH7* p.(Arg1712Gln) is a founder variant in a subset of French and Dutch patients.

## Introduction

Hypertrophic cardiomyopathy (HCM) is the most prevalent inherited cardiomyopathy, affecting 1 in 500 individuals in the general population. HCM is defined by hypertrophy of the left ventricular walls (≥ 15 mm in sporadic cases and ≥ 13 mm in the presence of family history) that is not explained by loading conditions. It is characterised by an increased risk of arrhythmias and sudden cardiac death (SCD).

A large number of genes have been associated with HCM, of which only 11 have definitive or moderate evidence to be associated with HCM [1]. The number of carriers of a given variant is usually small, and it is not easy to establish precise genotype-phenotype correlations for each variant, often making the individualised management of carriers challenging. Using clinical and molecular data from an international cohort, we aimed to delineate the clinical phenotype associated with the *MYH7* c.5135G > A p.(Arg1712Gln) variant, gain more evidence for its pathogenicity, and evaluate a potential founder effect.

## Methods

### Subjects

We retrospectively collected clinical and molecular data from 22 apparently unrelated probands who carried the p.(Arg1712Gln) variant in the *MYH7* gene (NM_000257.4) and 74 family members. Informed consent for publication was obtained from all patients or their legal representatives in accordance with the Declaration of Helsinki and national legal regulations.

### Molecular data

Analysis of cardiomyopathy-related genes was performed in 15 probands (68%) using targeted next-generation sequencing (NGS) analysis. The minimal NGS gene set included the following genes: *MYH7, MYBPC3, MYL2, TNNT2* and *TNNI3*. Single-gene analysis using Sanger sequencing and/or denaturing high-performance liquid chromatography of the following sets of genes was reported for probands evaluated before 2013: *MYH7* and *MYBPC3* (3 probands (14%)), *MYH7, MYBPC3, MYL2, MYL3, TNNI3, TPM1 *and *TNNT2* (3 probands (14%)), and *MYH7, MYBPC3, TNNI3, TPM1, TNNT2 *and *GLA *(1 proband (4%)). Cascade genetic testing was performed on family members when requested. Interpretation of variants was performed according to the American College of Medical Genetics and Genomics and the Association for Molecular Pathology (ACMG/AMP) guidelines [2] and the adapted ACMG/AMP criteria for *MYH7* [3].

### Clinical data

Clinical data were retrieved retrospectively from medical records. Clinical, electrocardiographic and echocardiographic/magnetic resonance imaging (MRI) data were collected for all patients. HCM was diagnosed according to the European Society of Cardiology HCM guidelines [4]. Particular attention was paid to the following clinical variables: age at HCM diagnosis, hypertension, atrial fibrillation, stroke, syncope, permanent pacemaker or implanted cardioverter-defibrillator (ICD) implantation and therapy, septal reduction therapy, cardiac transplantation, heart failure (HF), and family history of HCM or SCD. Age at HCM diagnosis was used as surrogate for penetrance [5].

### Haplotype analysis

Assessment of ancestry via haplotype analysis was conducted in 25 patients from the Netherlands and France. Haplotype data were available for 10 additional Dutch carriers for whom no clinical data were available, and they were not included in the cohort. We studied 15 dinucleotide repeat markers spanning an 8.7-Mb region on both sides of *MYH7* (see Table S1 in Electronic Supplementary Material). Protocols are available upon request.

To establish the phase of alleles, haplotypes were determined with co-segregation analysis of family members. When no family members were available, haplotypes were derived when the available allele matched a previously determined disease-associated haplotype as performed in a previous study [6].

## Results

### Molecular data

The *MYH7* p.(Arg1712Gln) variant was detected in all probands (*n* = 22). No other (likely) pathogenic variant was identified. Among the 73 family members who underwent cascade screening, 30 carried the *MYH7* variant. One additional family member (patient #8.17) was an obligate carrier by pedigree history.

### Clinical data

Phenotype data were available for 22 probands and 74 family members (Tab. 1 and Table S2).

**Table 1:** Clinical information of probands^a^

ID	Sex	AAD	ALE	Signs and symptoms at presentation	Maximum LV wall thickness	Left atrial dilatation	Obstruction of LV outflow	LVEF	AF	Events	FH of HCM	Variant identified in family member	FH of SCD
1	M	61	62	Chest pain and dyspnoea (61)	19–20 mm (IRM, 61); 19 mm (echo, 62)	Mild dilatation (echo, 62)	Yes, 13 mm Hg at Valsalva manoeuvre increasing to 30 mm Hg after extrasystole (62)	Normal (61) / 75.2% (62)	Yes (61)	Myocardial ischaemia under stress, coronary angiography: atheromatous arteries without significant stenosis (61)	No	No, none tested	U (35)
2	F	NA	84	NA	24 mm (echo, 72) 28 mm (echo, 84)	No, 19 cm^2^ (72) / yes, 29.3 cm^2^ (84)	Yes (72)	79% (72) / slightly altered (84)	No	No	Yes	No, none tested	NA
3	M	45	46	Pulmonary embolism	22 mm (echo, 45)	Yes, 437 ml/m^2^ (44)	NA	73% (44)	No	No	No	No	No
4	M	28	NA	NA	NA	NA	NA	NA	NA	No	Yes	Yes	No
5	M	16	37	Chest pain (14)	18 mm (MRI, 32); 25 mm (MRI, 37)	No	No	78%, (32)	No	NA	Yes	Yes	No
6	F	67	68	Heart murmur, dyspnoea, chest pain, dizziness	19 mm	Normal	Severe (6 m/s), SAM	Normal	Yes	No	Yes	Yes	Yes? (suspected in father)
7	F	68	76	Out-of-hospital cardiac arrest during exercise; VF	19 mm (MRI, 68)	Mild dilatation LA, borderline dimensions right atrium(68) / biatrial dilatation (76)	Midventricular obstruction with a dynamic systolic and diastolic gradient (76)	59% on MRI (68)	Yes (69)	VF (68), ICD (68), AF (69), persistent AF (75), repolarisation abnormalities (68)	Yes	Yes	Yes
8	M	48	63	Presyncope	19 mm (48); 20 mm	NA(48); mild dilatation(63)	No (48); no (63)	Normal	No	Some PVC (Holter)	Yes	Yes	No
9	M	51	51	Arrhythmias during exercise	NA	NA	NA	NA	No	Repolarisation abnormalities	Yes	Yes	No
10	M	29	71	NA	NA	NA	NA	NA	Yes	LBBB, pacemaker for asystolia and bradycardia (54), NSVT (59), ICD (59), ICD therapy (62), TIA (58), atrial flutter ablation (69)	Yes	Yes	M (30), S (18), B (40)
11	F	34	62	NA	18 mm (34)	NA	NA	NA	Yes	Borderline AV block, ablation for atrial flutter (62)	NA	No	No
12	F	74	76	None	21 mm (MRI, 74)	NA	NA	NA	NA	Intraventricular conduction delay	NA	No	NA
13	F	NA	72	NA	NA	NA	NA	NA	NA	Intraventricular conduction delay, ICD (72)	NA	NA	NA
14	M	40	40	None	16 mm	Dilated (45 ml/m^2^)	No	Normal	No	Borderline first-degree AV block (Holter)	No	No	F (48), U (42), GF
15	M	8	48	NA	18 mm (48)	NA	NA	NA	Yes	Myectomy (8), TIA (10), chronic heart failure (43), RBBB	NA	NA	NA
16	F	44	49	Chest pain, dyspnoea, dizziness, bradychardia	18 mm (44); 19 mm (echo, 49); 24 mm (MRI, 49)	NA	NA	NA	No	Septal reduction by myectomy (44), LBBB	NA	NA	NA
17	M	33	37	SVT	16 mm (echo, 33); 17 mm (echo, 37); 15 mm (MRI)	Normal (echo); dilated (MRI, 29 cm^2^)	NA / 13.5 mm Hg	NA / normal	No	SVT, ablation of posteroseptal accessory pathway (19 and 33)	No	No	No
18	M	66	71	NA	13 mm (66); 17 mm (71)	NA	NA	NA	No	NA	Yes	No	Yes (33)
19	F	NA	NA	NA	NA	NA	NA	NA	NA	NA	NA	NA	NA
20	M	NA	NA	NA	NA	NA	NA	NA	NA	NA	NA	NA	NA
21	F	60	76	NA	19 mm (echo, MRI)	NA	Slightly faster blood flow LVOT (61)	75% (70)	Yes (69)	Left anterior hemi block, tachycardia-bradycardia syndrome	Yes	No, none tested	NA
22	F	68	78	NA	17 mm (echo and MRI, 73); 14 mm (MRI, 78)	NA	Yes	Normal (73); 49% (78)	No	NSVT (78), aortic valve insufficiency, hypertension	Yes	No	No

*AAD* age at diagnosis, *AF* atrial fibrillation, *ALE* age at last evaluation, *AV* atrioventricular, *B* brother, *F* father, *FH* family history, *GF* grandfather, *ICD* implanted cardioverter-defibrillator, *IVS* interventricular septum, *LA* left atrium, *LBBB* left bundle branch block, *LV* left ventricular, *LVEF* left ventricular ejection fraction, *LVOT* left ventricular outflow tract*, M* mother, *MRI* cardiac magnetic resonance imaging, *NA* not available, *NSVT* non-sustained ventricular tachycardia, *RBBB* right bundle branch block, *S* sister, *SAM* systolic anterior motion, *SCD* sudden cardiac death, *SVT* supraventricular tachycardia, *TIA* transient ischaemic attack, *U* unknown family relation, *VF* ventricular fibrillation

^a^Age in years is shown between brackets

#### Probands

Of the 22 probands, 10 (45%) were females. Mean (± standard deviation) age at HCM diagnosis was 46.7 years (range: 8–74) in all probands, 38.6 years (range: 8–66) in males and 59.3 years (range: 34–74) in females. Mean age at last examination was 61.4 ± 19.3 years. Follow-up data were available for 77% (17/22) of the probands, with a median follow-up duration of 9 years after HCM diagnosis (range: 1–42).

At the time of diagnosis, mean maximal wall thickness was 17.5 ± 2.5 mm (range: 13–21), left ventricular outflow tract obstruction (LVOTO) was detected in 57% (4/7) of probands, and 2 additional probands had LVOTO but the age of onset was unknown. The most frequently reported initial manifestations were chest pain or tightness (5/11; 45%) and dyspnoea (3/11; 27%).

Patients #12 (male) and #14 (female) were asymptomatic at diagnosis. HCM was accidentally diagnosed in patient #12 at age 76 years during routine cardiologic evaluation following a fracture. Patient #14 was diagnosed with HCM at 40 years of age during cardiac screening for SCD family history. In 1 female proband (patient #7), the presenting symptom was SCD during moderate physical activity at age 68 years.

Atrial fibrillation was observed in 7/14 probands (41%). Two probands underwent septal reduction therapy, 2 developed a TIA, and 1 developed chronic HF. No HF deaths nor heart transplants were recorded. ICD implantation was reported for 3 probands at age 59 years in a male and at ages 68 and 72 years in 2 females.

A positive family history of HCM was reported for 79% (11/14) of probands. Five of 13 probands (38%) had a family history of SCD, occurring in 7 family members (2 females (29%)) at a mean age of 35 years (range: 18–48). Age at SCD and sex were unknown for 2 additional family members.

#### Family members

Of the 74 family members, 30 (14 females (47%)) tested positive for the familial p.(Arg1712Gln) variant and 1 was an obligate carrier. Of these 31 carriers, 16 (52%) were diagnosed with HCM, 6 (38%) of whom were females. Median age at HCM diagnosis in family members was 58 years (range: 20–72), and mean maximal wall thickness at the time of diagnosis was 16.5 ± 2.2 mm (range: 13–20). Follow-up data were available for 6 family members, with a median follow-up duration of 8.5 years after HCM diagnosis (range: 2–47). In genotype-positive relatives, the HCM phenotype varied widely, ranging from asymptomatic HCM to more severe disease requiring myectomy. None of the family members needed heart transplantation or died because of HF. Mean age at last examination of carriers without HCM was 42 ± 15.5 years (range: 21–64).

#### Penetrance

**Fig. 1:**
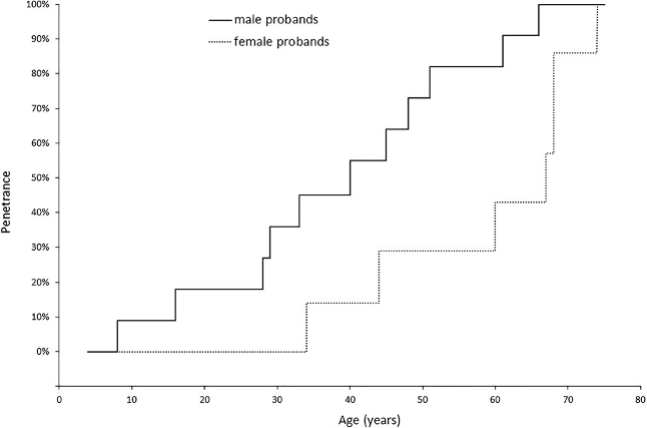
Inverted Kaplan–Meier curve indicating age-related penetrance of hypertrophic cardiomyopathy as survival variable in probands

**Fig. 2:**
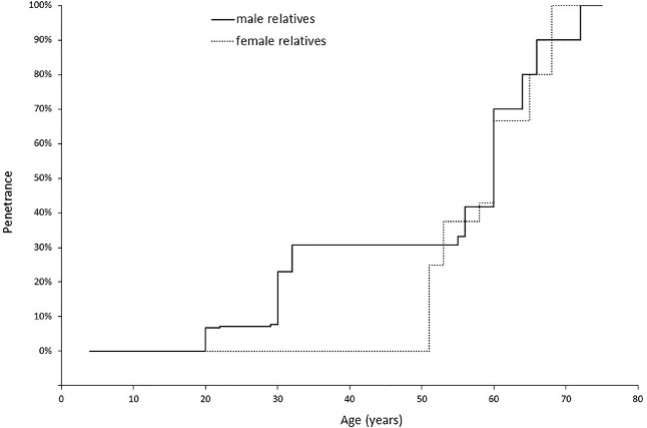
Inverted Kaplan–Meier curve indicating age-related penetrance of hypertrophic cardiomyopathy as survival variable in family members

Overall penetrance of HCM in our cohort was 72%, and this was sex- and age-dependent (Figs. 1 and 2). The earliest age at diagnosis was 8 years, and no women were diagnosed with HCM before age 34 years. Forty-four percent (14/32) of the individuals, half of whom were women, were diagnosed with HCM at age ≥ 60 years. While female probands were significantly older at HCM diagnosis than male probands (mean age: 59.3 ± 12.4 years vs 38.6 ± 18.2 years; *p* = 0.0063), there was no statistically significant difference in age at diagnosis between sexes among family members (mean age: 58 ± 17.7 years in women vs 49 ± 18.8 years in men; *p* = 0.1812).

### Haplotype analysis

DNA for haplotype analysis was available for 25 patients from 18 unrelated families (4 families from France and 14 from the Netherlands) and 10 additional Dutch carriers for whom we had no clinical data and who were not included in our clinical cohort (see Table S3 in Electronic Supplementary Material). We found 3 haplotype groups, which were all absent in relatives without the variant (data not shown). The largest group showed shared haplotypes of ≥ 4 markers, covering at least a 2-Mb region surrounding *MYH7* in 10 Dutch and all 4 French families, as well as in 8 additional Dutch carriers. Several recombination events occurred in this group, which are indicated by different colours in Table S3 in the Electronic Supplementary Material. In the second group, 3 Dutch patients shared haplotypes of ≥ 6 markers spanning a 2.2-Mb region.

## Discussion

We have described the phenotype associated with the *MYH7* p.(Arg1712Gln) variant in 22 probands and 31 family members, the largest cohort of individuals with this specific variant and the second largest cohort with a single *MYH7* variant to date. We have shown it is a founder variant with classical elements of inherited cardiac disease, i.e. clinical variability (yet a consistent HCM phenotype) and age-dependent penetrance.

Two major observations were made: the penetrance was overall delayed for both sexes compared with the *MYH7* subset from the HCM SHaRe registry (*n* = 492) [5], and no woman developed HCM before the fourth decade. In our cohort and in the *MYH7* subset of the HCM SHaRe registry, age at diagnosis was 58.8 ± 12.2 years and 34.8 ± 19.2 years in women (*p* < 0.0001), respectively, and 43.6 ± 18.7 years and 33.3 ± 16.8 years in men (*p* = 0.0028), respectively [5].

In the SHaRe registry, women and men developed HCM caused by different *MYH7* variants at similar ages (*n* = 492; 34.8 ± 19.2 years in women vs 33.3 ± 16.8 years in men; *p* = 0.39) [5], whereas we observed a statistically significant difference in age at diagnosis between sexes in our cohort as a whole (*n* = 53; 58.8 ± 12.2 years in women vs 43.6 ± 18.7 years in men; *p* = 0.0011) and in the probands (*p* = 0.0063). Nevertheless, there was no statistically significant difference in age at HCM diagnosis between sexes among family members (*p* = 0.1812), which could be explained by unknown genetic or clinical modifiers, a delay in reporting symptoms and/or a delay in cardiology referral in women who are not known to be at high risk of developing HCM (e.g. in the absence of a family history of HCM or SCD), as observed for other cardiac conditions [7–9].

In our cohort, only 1 patient was diagnosed with HCM before the age of 10 years. He underwent septal reduction by myectomy at 8 years of age and presented with chronic HF at the age of 43 years. Genetic screening of 23 HCM genes using NGS did not identify any additional disease-causing variant in this patient, but a second genetic HCM-causing variant that contributed to this early onset of the disease cannot be fully excluded.

Severity of HCM in our cohort varied largely, spanning from asymptomatic HCM to symptomatic and life-threatening arrythmias, with aborted SCD and HF reported in a female proband and history of SCD in 39% of the families. All relatives who did not carry the variant (*n* = 43) were not affected by HCM: 79% (*n* = 34) were evaluated using echocardiography and/or cardiac MRI, 12% (*n* = 5) were evaluated using electrocardiography only, and cardiac clinical screening was reported as normal in 9% (*n* = 4) without additional information.

The *MYH7* p.(Arg1712Gln) variant affects a highly conserved residue within the myosin tail domain and is predicted to be probably damaging to the protein structure/function PolyPhen‑2, deleterious by MutationTaster and by SIFT. This variant is present in gnomAD (http://gnomad.broadinstitute.org, accessed on 14 April 2023), with an allele frequency of 0.00002125 (6/282354). The 6 mutated alleles reported in gnomAD are all detected in the European, non-Finnish population at the heterozygous state in individuals who are in the 45–70-year age range. This variant is classified as pathogenic in the ClinVar database according to the ‘Expert Panel designation’ (https://www.ncbi.nlm.nih.gov/clinvar, accession number VCV000036642.42, accessed on 14 April 2023), and our data provide additional supporting evidence for its pathogenicity. Although it has been reported to be associated with HCM in the literature (Tab. 2), as far as we know we are the first to prove its founder effect.

**Table 2:** Overview of carriers of *MYH7* p.(Arg1712Gln) variants reported in literature

Number of patients	Phenotype	Additional informations	Reference
1	HCM	Childhood-onset, familial HCM	Morita, et al. N Engl J Med. 2008;358:1899–908
1	HCM	44-year-old female; apical aneurysm, MWT 14 mm, ECG abnormalities, negative T wave; positive family history; also carrried *TNNI3* variant p.(Arg162Gln) (gnomAD allele frequency 10/249030)	Gruner, et al. Circ Cardiovasc Genet. 2011;4:288–95
4	Unknown	4 unrelated individuals from published data, a reported clinical cohort, and clinical laboratory data provided in genetic test report	Pan, et al. Circ Cardiovasc Genet. 2012;5:602–10
1	HCM	Age at diagnosis: 19 years	Miller, et al. J Genet Couns. 2013;22:258–67
1	HCM	None	Mook, et al. J Med Genet. 2013;50:614–26
7	HCM?	Study on 427 unrelated, ostensibly healthy individuals from various racial and ethnic backgrounds and HCM case cohort of 2178 individuals	Kapplinger, et al. J Cardiovasc Transl Res. 2014;7:347–61
3	Asymptomatic	Asymptomatic, pre-hypertrophic mutation carriers	Witjas-Paalberends, et al. Cardiovasc Res. 2014;103:248–57
1	HCM	None	Lopes, et al. Heart. 2015;101:294–301
3	Cardiomyopathy	3 patients from Russia/Belarus	Glotov, et al. Clin Chim Acta. 2015;446:132–40
7	HCM	None	Homburger, et al. Proc Natl Acad Sci U S A. 2016;113:6701–6
3	HCM	1 patient also carried *MIB1* c.2530_2532delTCTinsC variant	Van Velzen, et al. Am J Cardiol. 2016;118:881–887
1	HCM	36-year-old male; myecotomy; MWT 17 mm	Helms, et al. Circulation. 2016;134:1738–48
16	HCM	Variant found in 16/6112 HCM patients	Walsh, et al. Genet Med. 2017;19:192–203
1	HCM	Patient possibly already described in Gruner, et al. Circ Cardiovasc Genet. 2011;4:288–95	Weissler-Snir, et al. Circ Cardiovasc Imaging. 2017;10(2). pii: e005311
3	HCM	Patients also reported in our cohort	Van Lint, et al. Neth Heart J. 2019;27:304–9
1	HCM	82-year-old Spanish female	Mademont-Soler, et al. PLoS One. 2017;12:e0181465
1	HCM	Also carried *MIB1* c.2530_2532delTCTinsC; patient probably already reported in Van Velzen, et al. Am J Cardiol. 2016;118:881–7 and Van Velzen et al. Am J Cardiol. 2018 Sep 8	Van Velzen, et al. Circ Cardiovasc Genet. 2017;10:e001660
35	HCM	17 published probands and 18 private (= laboratory internal data) probands	Kelly, et al. Genet Med. 2018;20:351–9
1	HCM	None	Mak, et al. Sci Rep. 2018;8:10846
2	HCM	2 male patients: 1 also carried *MIB1* c.2530_2532delTCTinsC, another also carried *MYH7* variant c.3100-2A > C; both were probably already reported in Van Velzen, et al. Am J Cardiol. 2016;118:881–7	Van Velzen, et al. Am J Cardiol. 2018;122:1947–54
1	HCM	43-year-old Vietnamese patient; chest pain, eccentric LV hypertrophy, maximal LV thickness 27 mm	Tran Vu, et al. Circ J. 2019;83:1908–16
≥ 1	HCM	None; patient(s) already reported?	Thomson, et al. Genet Med. 2019;21:1576–84
1	HCM	32-year-old male	Cao, et al. Stem Cell Res. 2021;55:102455

*ECG* electrocardiogram, *HCM* hypertrophic cardiomyopathy, *LV* left ventricular*, MWT* maximal wall thickness

### Implications

This study showed that studying a large series of individuals with a single specific variant underlying an inherited cardiac disease may lead to observations different from the general picture associated with a specific disease or its underlying gene or variant, as we have shown previously for other genes/variants [10]. Therefore, we propose a variant-specific approach for *MYH7* p.(Arg1712Gln) carriers.

Current guidelines recommend precautionary regular cardiac evaluations in healthy carriers of HCM-causing variants as the disease may appear late in adulthood [11, 12]. Clinical screening of healthy carriers is usually performed every 1–3 years in childhood and every 2–5 years in adulthood, according to national and international guidelines [4, 13, 14]. Since no women in our cohort were diagnosed with HCM before age 34 years, longer intervals between follow-up examinations could be considered for women < 30 years with this specific variant.

It remains difficult to establish the age at which to discontinue cardiac follow-up in genotype-positive, phenotype-negative individuals. According to French national guidelines, clinical follow-up is usually stopped in healthy carriers > 60 years, when the risk of developing the disease is considered to be low [13], whereas a Dutch national guideline working group states that there are insufficient data to establish an age limit for long-term follow-up of healthy carriers of HCM-causing variants [14]. Since 6/13 probands (4 women) and 4/31 family members (1 woman) were diagnosed with HCM after the age of 60 years, we suggest that cardiac follow-up of healthy carriers of the *MYH7* p.(Arg1712Gln) variant should be continued after the seventh decade.

### Study limitations

The limitations of our study include its retrospective nature and the non-standardised assessment of patients since clinical information was obtained from patient files from multiple centres across 2 countries. Moreover, limited clinical data were available for a number of patients.

## Supplementary Information

**Table S1** Dinucleotide repeat markers

**Table S2** Clinical information of family members

**Table S3** Haplotype results
