# Supplementary material for: MYH7 p.(Arg1712Gln) is pathogenic founder variant causing hypertrophic cardiomyopathy with overall relatively delayed onset
Source: Neth Heart J. 2023 Jul 24;31(7-8):300–7. doi: 10.1007/s12471-023-01798-9 (PMC10400741; doi:10.1007/s12471-023-01798-9)
Supplement: Supplementary file 2 — Table S2 Clinical information of family members [file 12471_2023_1798_MOESM2_ESM.docx]

**Tab. 2**. Clinical information of family members^a^

| **ID** | **Carrier** | **Sex** | **AAD** | **First evaluation** | **Follow-up evaluation** |
| --- | --- | --- | --- | --- | --- |
| 1.1 | Yes | M | - | Unaffected (NA) | NA |
| 4.1 | Yes | M | 32 | HCM (32); IVS: 10.5 mm (echo, 27), 5 mm (echo); LVOTO at effort; normal ECG; no AF | NA |
| 4.2 | Yes | M | 66 | HCM (66); IVS: 18 mm (MRI); moderate LA dilatation (echo); ECG: negative T wave in inferior leads; no LVOTO; no AF | NA |
| 4.3 | No | M | - | IVS: 14 mm (echo, 74), hypertension, diabetes | - |
| 4.4 | Yes | F | - | Unaffected (59) | - |
| 5.1 | No | M | - | Unaffected (71) | - |
| 5.2 | No | M | - | Unaffected (46) | - |
| 5.3 | Yes | M | 64 | HCM; IVS: 13 mm (echo); LAE: 21 cm^2^, 41 ml/m^2^; LVEF: 69% | NA |
| 5.4 | No | M | - | Unaffected (19) | - |
| 5.5 | No | F | - | Unaffected (18) | - |
| 5.6 | Yes | F | - | Unaffected (33) | - |
| 5.7 | No | F | - | Unaffected (40) | - |
| 5.8 | No | F | - | Unaffected (21) | - |
| 5.9 | Yes | M | 72 | HCM (72); IVS: 16 mm (echo); no LVOTO; no AF; no ECG abnormalities | NA |
| 6.1 | No | F | - | Unaffected (76) | - |
| 6.2 | No | F | - | Unaffected (71) | - |
| 6.3 | No | F | - | Unaffected (66) | - |
| 6.4 | No | F | - | Unaffected (64) | - |
| 6.5 | No | F | - | Unaffected (63) | - |
| 6.6 | No | M | - | Unaffected (43) | - |
| 6.7 | No | F | - | Unaffected (33) | - |
| 6.8 | Yes | F | - | Unaffected; chest pain on exertion, ST segment elevation in V1 and sinus bradycardia; no abnormalities on echo/exercise ECG (42) | - |
| 6.9 | Yes | F | - | Unaffected (64): left axis deviation of heart, no other abnormalities on echo/ECG | - |
| 6.10 | Yes | M | 60 | HCM (60); IVS: 13 mm | NA |
| 7.1 | No | F | - | Unaffected (39) | - |
| 7.2 | No | F | - | Unaffected (68) | - |
| 7.3 | No | M | - | Unaffected (38) | - |
| 7.4 | Yes | M | - | Unaffected (21) | - |
| 7.5 | No | F | - | Unaffected (18) | - |
| 7.6 | No | F | - | Unaffected (55) | - |
| 7.7 | Yes | F | - | Unaffected (37); mild LAE (echo); no abnormalities on ECG | - |
| 7.8 | Yes | M | 56 | HCM; IVS: 15 mm, AF requiring cardioversion (56) | NA |
| 7.9 | Yes | M | - | Unaffected (54); no LVH on echo/MRI; no abnormalities on ECG/exercise ECG/Holter (except for sporadic PVC at Holter); palpitations | - |
| 8.1 | Yes | F | - | Unaffected (23) | - |
| 8.2 | Yes | F | - | Unaffected (27) | - |
| 8.3 | Yes | M | - | Unaffected (28) | - |
| 8.4 | Yes | F | - | Unaffected (30) | - |
| 8.5 | Yes | F | 51 | HCM (51); IVS: 18 mm; palpitations | NA |
| 8.6 | No | F | - | IVS: 13 mm (concentric), no abnormalities on ECG/exercise ECG (68); hypertension | - |
| 8.7 | No | F | - | Unaffected (66) | - |
| 8.8 | No | M | - | Unaffected (63) | - |
| 8.9 | No | M | - | Unaffected (61) | - |
| 8.10 | No | F | - | Unaffected (58) | - |
| 8.11 | No | M | - | Moderate concentric LVH (echo), repolarisation abnormalities (ECG), negative exercise ECG (56); hypertension | - |
| 8.12 | No | F | - | Unaffected (52) | - |
| 8.13 | No | M | - | Unaffected (50) | - |
| 8.14 | No | F | - | Unaffected (49) | - |
| 8.15 | Yes | F | - | Unaffected (57) | - |
| 8.16 | Yes | M | - | Unaffected (59); repolarisation abnormalities (ECG); normal heart morphology (echo); pre-syncope | - |
| 8.17 | Obl | F | NA | HCM (NA); deceased (80) | NA |
| 8.18 | Yes | F | 60 | HCM (60); IVS: 17 mm, palpitations, pre-syncope, fatigue | NA |
| 8.19 | Yes | F | 68 | HCM, IVS: 18 mm (68) | IVS: 18 mm (73); LVOTO 15 mmHg (73) |
| 9.1 | Yes | M | 20 | NA | IVS: 16 mm (echo, 54); IVS 17 mm (57); LVOTO 8 mmHg, no SAM (66) |
| 9.2 | No | M | - | IVS: 15–18 mm, concentric (echo, 53); malignant hypertension | - |
| 10.1 | Yes | M | 30 | Normal MRI (22) | IVS: 20 mm (30); ICD implant, primary prevention (30); SVT (32); LVOTO 245 cm/s and SAM (34) |
| 10.2 | Yes | M | 60 | HCM (60); IVS: 19 mm; palpitations | NA |
| 10.3 | No | M | - | Unaffected (72) | - |
| 10.4 | No | M | - | Unaffected (70) | - |
| 10.5 | No | F | - | Unaffected (70) | - |
| 10.6 | No | F | - | Unaffected (68) | - |
| 10.7 | No | M | - | Unaffected (64) | - |
| 10.8 | No | M | - | Unaffected (68) | - |
| 10.9 | No | F | - | Unaffected (71) | - |
| 10.10 | No | F | - | Unaffected (61) | - |
| 10.11 | No | F | - | Unaffected (59) | - |
| 10.12 | No | F | - | Unaffected (59) | - |
| 10.13 | No | F | - | Unaffected (58) | - |
| 10.14 | No | F | - | Unaffected (60) | - |
| 10.15 | No | F | - | Unaffected (48) | - |
| 10.16 | No | ? | - | Unaffected (46) | - |
| 10.17 | Yes | F | 53 | HCM (53) | NA |
| 22.1 | Yes | M | 30 | HCM (30) | Myectomy (55), died of unknown cause (55) |
| 22.2 | Yes | M | - | Unaffected (56); IVS 12 mm NSVT(55); Holter: 3x doublet |  |
| 22.3 | Yes | F | NA | NA | HCM: IVS: 13 mm (MRI, 51), RBBB (ECG, 49) |

^a^ Age in years is shown between brackets

*AAD* age at diagnosis, *AF* atrial fibrillation, *echo* echocardiogram, *ECG* electrocardiogram, *ICD* implanted cardioverter-defibrillator, *IVS* interventricular septum, *LA* left atrial, *LAE* left atrial enlargement, *LVEF* left ventricular ejection fraction, *LVH* left ventricular hypertrophy, *LVOTO* left ventricular outflow tract obstruction, *MRI* cardiac magnetic resonance imaging, *NA* not available, *NSVT* nonsustained ventricular tachycardia, *Obl* obligate carrier, *PVC* premature ventricular contraction, *RBBB* right bundle branch block, *SAM* systolic anterior motion of anterior mitral valve leaflet
